# Applying the integrated marketing communication approach to recruit and retain African American women

**DOI:** 10.34172/hpp.2021.58

**Published:** 2021-12-19

**Authors:** Traci Hayes, Manoj Sharma

**Affiliations:** ^1^Department of Public Health, School of Health Professions, University of Southern Mississippi, Hattiesburg, USA; ^2^Department of Social and Behavioral Health, School of Public Health, University of Nevada, Las Vegas, USA

**Keywords:** Health communication, Health promotion, Social marketing, Exercise, African Americans

## Abstract

**Background:** Researchers use multiple approaches to engage and maintain underrepresented populations in research. They often overlook integrated marketing communication (IMC), a useful approach for commercial marketing, for more established health promotion and social marketing techniques. There is limited information on the application of the IMC approach for recruiting and retaining African American study participants. This article explores the IMC approach used to recruit and retain volunteers for a community-based intervention.

**Methods:** This is a cross-sectional study relying on extracted data from the Multi-Theory Model (MTM) of Health Behavior Physical Activity intervention. A brief multiple-choice survey was administered to a sample of African American women (n=74) to assess the effectiveness of applying an IMC approach for recruiting and retaining volunteers for the multi-week program during January - June 2018. The measures were (1) source for study information, (2) preferred method of contact, (3) primary source for health information.

**Results:** Sixty-nine women listed their doctor as the primary source of health information and five women in the age group 18-34 identified social media (n=3) and websites (n=2). Age is significantly related to the preference of communication tools used to recruit and retain the African American participants. A statistical significance (*P* =0.025) suggests for women ages 51-69, a combination of radio, church, and word of mouth was more effective for recruitment. The older women preferred telephone calls compared to the women ages 18-50 who relied on texting.

**Conclusion:** IMC can synergize individual communication elements in a coordinated manner to address niche audiences and develop cost-effective health communications programs that can improve recruitment and retention efforts in minority populations.

## Introduction

There is growing interest in recruiting and retaining individuals from diverse racial and ethnic groups for research studies and trials.^1-3^ It is important to include diverse groups in clinical research because it improves the applicability of the research findings to their real-world environment.^4-7^ The process of recruiting and retaining minority groups is often arduous.^5,8^ Healy and colleagues^3^ posits that less than 50% of the studies meet the recruitment goals and retention is difficult. Research suggests that training to conduct the intervention does not always translate into successful recruitment and retaining participants.^9,10^ Graham et al^11^ found that misunderstandings due to poor communication and that the lack of public information about research opportunities were the contributing factors for poor participation, particularly among African American women. Many recruitment and retention challenges such as lack of interest and motivation, time constraints, social demands, as well as haircare maintenance have been identified.^5,6,8,10,12^

Researchers use multiple approaches to engage and maintain underrepresented populations in research.^10,13-15^ Many recognize mass media as an effective resource to form and change attitudes towards new health behaviors.^1^ In their evaluation of traditional and online promotional strategies, Bracken and colleagues found that targeted radio advertising (42%), coverage in television news (20%), and mass mailouts (17%) were the most effective for recruiting men to participate in a multi-center diabetes prevention trial.^13^ More than 19 000 men were screened and 1007 (5%) were enrolled in the study. News, radio, posters, newspaper advertisements, participation at local events, online promotions, Google advertising, and Facebook advertising each accounted for less than 4% of those enrolled. The most cost-effective paid strategy was mass mailouts. The authors suggested four key principles, namely, specific target audience, concerted call to action, relevant promotional content, and effective platforms for promotion.^13^ In their review to examine the traditional (television, print ads, and radio) approaches compared to innovative (internet, and social media) approaches for smoking quitlines, Momin and colleagues^16^ found the internet recruited younger participants across all studies. Effective media strategies accounted for 35%-54% of the enrolled volunteers. In one study, the newspaper was the primary source, followed by radio, newsletters, and television. According to authors, studies using mass mailing had low participation and the studies relying on posters in targeted locations and presentations found the methods ineffective, accounting for just 2% to 4% of the participants.^16^

Im and colleagues^14^ suggest researchers use multiple recruitment sources to reach racial and ethnic minorities. Some of the literature suggests that researchers need to be flexible and apply traditional and non-traditional ideas to achieve recruitment and retention goals. ^1,11,13,14,17^ According to Beech and colleagues, recruitment approaches lack theoretical grounding and sufficient tailoring to targeted populations for adequate enrolment and retention of minority groups.^1^ There is limited information on the application of the integrated marketing communication (IMC) approach for recruiting and retaining study participants.

Dahl et al^18^ state that IMC is a useful approach for promoting specific health programs but overlooked for more established health promotion and social marketing techniques. IMC is the science of aligning a variety of interactions to deliver a targeted, concise message to the consumer. The IMC approach coordinates a centralized message across multiple communication channels and encompasses media such as blogs, webinars, radio, television, and print. According to Drewnowski et al,^2^ not all populations may use or may have equitable access to media (social or mass), and therefore multiple channels may be required. Our research question was to determine whether the IMC approach is an effective method for recruiting and retaining participants in a community-based intervention.

### Integrated marketing communications

Graham et al^11^ proposed there are two approaches to recruitment (1) using a multitude of active and diverse strategies and (2) traditional approach, utilizing fewer and more passive strategies. Applying IMCs aligns with the approach to utilize various and diverse strategies to recruit and retain. According to the research, an IMC approach can be advantageous when utilized in health communication efforts.^17-23^

IMC involves the coordination of convincing and purposeful messages that target the end-users.^23^ The media works in tandem as one form of communication to drive the individual to access other media to acquire complementary information.^17^ IMC relies on multiple channels while the message remains consistent and drives attention, interest, desire, and action (AIDA).^21,22^ IMC approach infused with AIDA-focused content was disseminated through multiple communication channels working in tandem. The message aimed to:

1. Attract attention to the health issue or program (participating in an intervention to initiate and sustain physical activity),
2. Raise interest based on benefits and value-added (opportunity to improve your health and to have access to the other incentives provided for volunteering),
3. Convince volunteers that they wanted to be involved (message reinforcement via multiple channels), and
4. Take steps to engage or participate (clear instructions and steps to screening and enrolment).

African Americans remain underrepresented in health promotion programs and fail to experience the benefits of the interventions that show promise for addressing some health conditions.^1,3,4,11^ The Centers for Disease Control and Prevention (CDC) acknowledged the usefulness of the IMC approach as a social marketing and health communication resource, but it remains unused for most health promotion efforts.^21^ There continues to be a need for strategies that prompt AA participation, and the IMC has characteristics that can improve efforts to communicate with AA and thus secure their engagement in health promotion research. This study describes the use of the IMC approach underpinning the recruitment and retention efforts for a community-based intervention and seeks to contribute practical insight for employing multiple communications channels to bolster participation from African American women.

This article explores the IMC approach used to recruit and retain volunteers for a community implemented, theory-based physical activity intervention, Multi-theory Model (MTM) of Health Behavior Change Physical Activity Intervention.^24^ In this article, we discuss how traditional and digital modes of communication were coordinated to recruit and maintain African American women in the randomized controlled trial.

## Materials and Methods

The current study relies on extracted data from the Multi-Theory Model of Health Behavior Physical Activity intervention which was a randomized controlled trial (Protocol #0092-17), conducted between January – June 2018, at the Walter Payton Recreational Center at Jackson State University, and targeted African American women in Central Mississippi. A detailed description of the MTM physical activity (PA) intervention setting and study criteria has been published.^24^ The current study focuses on the MTM physical activity intervention recruitment and retention plan anchored by the IMCs approach, a framework that leverages multiple channels to deliver a centralized message. The application and effectiveness of the IMC approach is the focus of this current article.

### Study participants

The MTM intervention sample size was a priori; calculated using G*Power (with an alpha of 0.05, a power (1-beta) of 0.80, and effect size f = 0.30) requiring 40 individuals to achieve statistical significance.^24,25^ The effect size of 0.30 is widely accepted for social and behavioral sciences.^25^ Seventy-four adult women who (1) resided in Central Mississippi, (2) were ages 18-69 years of African American or African ancestry, and (3) attended the screening for the MTM PA intervention held on January 19, 2018, are the subjects of the current article. These women were exposed to the study recruitment announcements via public access television, radio, social media, email, outreach, and word-of-mouth (i.e., publicly displayed signage or from a third party).

### Study design

The cross-sectional study relies on extracted data from the MTM intervention. The data for the current research were collected using a brief survey comprised of four questions; three multiple choice and one dichotomous question were answered during the screening (orientation day) for the physical activity intervention.^24^ The participants were asked to select an answer for each of the questions, “Identify how you heard of the physical activity study”, “Did you hear about the physical activity study from more than one source”, “Identify your preferred method of contact to receive study updates and announcements”, and “Identify your primary source for health information”.

### Recruitment strategy

Relying on the IMC approach, the recruitment efforts combined various channels including (1) public access television, (2) community radio, (3) church outreach, (4) e-mail campaign, (5) social media and (6) publicly displayed flyers to promote a centralized, targeted message.

*Public access television*

A 60-second public announcement aired on the City of Jackson’s Public, Education, and Government (P.E.G.) Network (Channel 18). The TV public service announcement (PSA) aired during evening programming two times per week from November 2017 – January 2018. The P.E.G. Network Channel 18 is available to more than 2 0000 Comcast Xfinity customers in the Jackson and surrounding areas. Donated by the television station were the production costs and airtime, a $500.00 value.

*Community radio*

Radio promotion consisted of a 30-second announcement that aired twice a day, Monday – Friday, from December 2017 through January 2018, on WMPR-90.1. In addition to the daily radio announcements, the principal investigator discussed the study on the station owner’s weekly talk show. The cost of the production and radio announcements was $400.00.

*Church outreach*

The church outreach involved leveraging existing relationships and included three area churches that participated in a previous cross-sectional study of the multi-theory model to predict physical activity.^26^ Secondly, pastors at seven churches near the study site location received the study flyer and a letter via e-mail and a follow-up call. Two churches invited the principal investigator to present the study information to the lady auxiliaries. Church outreach continued from November 2017 through January 2018. The cost was minimal. The total of $25.00 covered printing, postage, and transportation to the churches.

*E-mail campaign*

Approval was received from the University to utilize a listserv of e-mails for the undergraduate and graduate students who were actively enrolled during the Fall semester 2017. The study flyer was emailed to approximately 3091 females at the university. The e-mail was disseminated once every two weeks from late November 2017 through January 2018. There was no cost ($0) associated with the email distribution.

*Social media*

The electronic version of the study flyer was posted to Facebook for public viewing. The study announcement was reposted once a month between November 2017 through January 2018. There was no cost ($0) associated with posting the flyer on Facebook.

*Word-of-mouth*

Study flyers and signage were posted in public locations. Information was handed to African American women at random in high-traffic locations. The total of $50 covered the printing costs and supplies needed for posting 100 flyers.

### Retention strategy

The IMC approach was applied to the retention strategy. Complementing resources were used to reinforce the study message and to ensure reciprocal engagement. The African American women who met the inclusion requirements were enrolled in the intervention. Retaining study participants was vital to the success and validity of the study. Like recruiting, retention has its own challenges.^1,3,6,11,27,28^ The IMC approach served as the framework for retention efforts. Email, phone, and text messages as identified by the participants were the preferred methods for communicating study updates. Motivational and instructional communications sent once a week via the participants’ identified preferred method of communication. The weekly communication were included a link to the study website that served as an archival hub for study information. The participants were able to reply to posts. The weekly communications and study website were used for ensuring continued participation and engagement.

### Statistical analyses 

The statistical analyses were conducted using IBM SPSS Version 25 (https://www.ibm.com). Descriptive statistics including frequencies and percentages were used to explain findings. A chi-square test of independence was used to investigate whether there was an association between age groups and selection of recruitment strategy and preference of retention strategy. Statistical significance was set at alpha = 0.05. We did not control for confounding variables in the current study.

## Results

Initial contact between potential volunteers resulted in 74 African American women who attended the MTM intervention orientation. The demographic characteristics for the seventy-four (n = 74) women who completed the communication survey are presented in Table 1.

**Table 1:** Demographic characteristics of all participants (n = 74)

**Characteristics**	
Age, Mean (SD)	45.93 (16.40)
Education, No. (%)	
High school graduate	8 (10.8)
Some college	19 (25.7)
College graduate	25 (33.8)
Graduate or professional degree	21 (28.4)

The women completed a brief survey to identify which communication source made them aware of the study recruitment opportunity, preferred modes of communication (retention), and source of health information. Sixty-nine women listed their doctor as the primary source of health information and the remaining five women in the age group 18-34 identified social media (n = 3) and websites (n = 2). The results of the selected recruitment strategy by age are presented in Table 2.

**Table 2:** Selected recruitment strategy by age (n = 74)

**Demographics**	**18-34**		**35-50**		**51-69**		***P*** ** value**
	**No.**	**%**	**No.**	**%**	**No.**	**%**	
Strategy							
Radio	2	2.7	6	8.1	8	10.8	0.025
Television	0	0	0	0	1	1.4	
Church	2	2.7	5	6.8	6	8.1	
Word-of-mouth	3	4.1	7	9.5	10	13.5	
Social Media	2	5.4	5	6.8	4	5.4	
E-mails	6	8.1	4	5.4	1	1.4	

The frequencies and percentages of the preferred retention tools by age groups are presented in Table 3.

**Table 3:** Preference of retention strategy among participants by age (n = 74)

**Demographics**	**18-34**		**35-50**		**51-69**		***P*** ** value**
	**No.**	**%**	**No.**	**%**	**No.**	**%**	
Strategy							
Telephone	1	1.4	4	5.4	13	17.6	0.012
Text messages	13	17.6	22	29.7	14	18.9	
E-mail	3	4.1	1	1.4	3	4.1	

The results of the chi-square test for the selection of recruitment approach by age were statistically significant (*P*= 0.025) suggesting that there was a relationship between the type of recruitment communication and participant age groups with older women preferring radio, church, and word of mouth. There was also a significant difference between the age groups and the retention tools with older women preferring telephone while middle-aged and younger women preferring text messages (*P* = 0.012).

## Discussion

Relying upon the IMC approach of utilizing multiple communication and outreach to disseminate a central message, the MTM-based physical activity study recruitment and retention goals were met.^24^ The purpose of this article was to discuss the application of the IMC approach used for recruiting African American women as volunteers for a randomized physical activity study. Approximately 114 women contacted the study coordinator. Seventy-four of those women attended the orientation to complete the screening process and a brief survey that provided the data for the current study. The women identified how they were informed of the study and whether they had been exposed to more than one recruitment source for study information.

### Recruitment

Optimal communication requires a suitable messenger who can provide a persuasive message to the priority audience at the most appropriate time.^26^ Word-of-mouth is an effective approach because it is “the best advertisement” for both genders and all age-groups.^8^ The direct exchange of information from a trusted source and individuals responding to the information posted in various community locations were the word-of-mouth recruiting strategies.^8^ For this study, word-of-mouth was selected as the primary source for study information. Word-of-mouth included information presented directly to the individual by a study stakeholder or community member and through flyer postings in high-traffic locations.

Chang and colleagues found the flyer was ineffective and had to revamp its message and relied on individuals at specific sites to create rapport and deliver information to potential volunteers.^6^ According to James and colleagues, churches are favorable environments for recruiting because the information shared in the setting is considered credible and beneficial.^8^ Churches are a source for repeat participants as well.^8^ Fam and Ferrante recommended someone to serve as a “champion” to facilitate communication and recruitment strategies.^7^ Beech et alsuggest an influencer or champion who can recruit at least 10 others to help meet the study recruitment goal.^1^ Fam and Ferrante presented several recommendations for recruitment that included someone to serve as a “champion” along with ongoing communication with staff, flexible participation time, reinforced confidentiality, and offering incentives.^7^ The researchers suggest planning for longer recruitment time.

The frequency of messaging disseminated via the local black community radio station proved an effective medium and at a lower price than the leading market radio stations. Bracken and colleagues found targeted radio ads as the most effective recruitment strategy for their multi-center diabetes study.^13^ The researchers utilized multiple traditional and online tools. More than 20% of the respondents learned of the RCT opportunity through radio exposure. O’Hara found that volunteers recruited via mass media such as radio and TV were more likely to enroll in the Get Healthy study.^15^ Only one participant in the current study acknowledged the PSA as a source of information about the MTM PA intervention recruitment opportunity. Approximately 18% of the participants selected social media (13) and another 18% selected the church visits (13), both were viable sources for recruiting participants. Word-of-mouth was the source of study participation information for 27% of the participants.

Thompson promotes social media’s usefulness for educating and engaging participants in clinical trials and can serve as a hub of communication.^27^ Facebook reaches more people than text and e-mail.^8^ Social media provided a free platform to promote. Facebook members reposted the flyer. The internet has expanded the pool of potential participants and accessibility to hard-to-reach volunteers.^14^ According to Graham and colleagues, mass mailings, health fairs, churches, and wellness centers were more suitable for recruiting participants in clinical research studies.^11^ Beech and colleagues^1^ delivered presentations at two annual community events to help fuel longstanding interest in their study. The process of engaging with the local churches included building upon existing relationships and sending mailers to churches without existing contact.^26^ Two churches responded to the mailers and the team delivered two separate presentations about the study and recruitment opportunities. The church buy-in may have spurred word-of-mouth outcomes. Many of the women who attended the church presentations expressed wanting to help and may have by sharing the study information with others.

Johnson and colleagues suggested meeting the volunteers in their own environment and acknowledged the importance of community support for engaging minorities.^10^ Im et al^14^ posited that targeted correspondence and personal meetings worked better than radio and television. Bracken et al^13^ found that mass mailouts were the most cost-effective strategy. Mass e-mails were disseminated via the university e-mail system to reach women 18 years and older at the undergraduate and graduate levels. Mass e-mails were sent to 3,114 students. Eleven volunteers (15%) indicated the e-mails as the source of their study awareness.

Results of the selection of recruitment approach by age were statistically significant (*P* = 0.025) with older women preferring radio, church, and word of mouth. Future recruitment efforts directed toward older African American women must keep these approaches in mind.

### Retention

Three communication methods (text, phone call, and e-mail) were available to study participants for receiving study information and updates. Participants were informed of the ways to acquire study information and updates and communicate concerns and issues pertaining to the study. The study contact number and the website were provided to all the participants.

The current study participant wanted weekly reminders and updates via text (n = 49; 66%). The participants received weekly follow-up via the communication method that they identified. James et al^8^ recognized that most participants had smartphones. Hall et al^29^ found that text messaging was effective in influencing health outcomes and behaviors. Text messaging was immediate and customizable, and transmitted critical information that could be referenced later and typically was less time consuming.^26^ Some study participants selected phone (n = 18; 24%). Chang et al^6^ note a high degree of participant failures to respond to phone calls from the researchers. During the current study, weekly phone calls were placed and often many were answered. The high call contact may be due to sharing the study phone number and encouraging participants to save the information as a contact in their phones. Valentiner et al^23^ found that follow-up telephone calls helped to improve adherence in their study. For the current study, enabling the participants to direct the method by which they received information resulted in frequent contact and interaction between the study coordinator and participants.

There was also a significant difference between the age groups and the retention tools with older women preferring telephone while middle-aged and younger women preferring text messages (*P* = 0.012). This finding implies that to retain older women, the traditional approach of calling on telephones will be an important tool for future studies. Younger and middle-aged African American women are more amenable to text messaging.

### Study website 

The study website/blog aided in sharing and archiving information and updates. According to some researchers, digital media such as blogs serve as an additional source for collaboration, customizing, and focusing the message.^27,30^ The website linked participants to information and provided a platform to share views and experiences of study engagement. The website URL and study contact information was included in all information.

Limitations of the research included focusing on study data that only addressed African American women interested in the physical activity study and does not represent other groups. Therefore, the outcomes may not be generalizable or expected when recruiting and retaining other populations. The data collection was limited to only a few variables. This study was descriptive in that it focused on collecting data on a limited number of variables and did not control for any confounders or rely on sophisticated statistical data analyses. Though the intervention aimed at sustaining the health behaviors, there was no implementation of follow-up communications program to monitor the long-term behavior change. In many cases, targeting specific groups such as African American women only may not be possible when incorporating mass media and will require consideration as to how to deliver a socially appropriate recruitment message. The study focuses on the recruitment and retention of a small sample; applying the IMC approach to a larger sample of participants may generate different results.^31^ The financial and human resources were limited and could support only a minimal number of participants.

Despite having relatively low participation given the frequency of message dissemination, the integration of the media achieved reach and impact to secure volunteers from various age groups and backgrounds during the short recruitment period. The IMC approach demonstrated its usefulness in combatting recruitment barriers such as lack of interest, motivation, and knowledge by ensuring the study information was accessible through multiple channels. These efforts reinforced the relevance and benefits of participation amongst a population that is traditionally hard to engage and retain. Centralizing messages through their preferred modes of communication allowed for the delivery of reminders to prompt action from an “overly committed” audience and demonstrated that the researchers’ were willing to adapt to the participants’ need for flexibility and continual follow-up.

### Future research

Future studies should explore the effectiveness of the IMCs approach for recruiting larger sample of the minority population. Second, the research should determine the association of specific recruitment tools with enrollment. Participants should discuss their recruitment and retention experience to identify strategies for the successful engagement of minority populations.^7^ Future studies can inform approaches for future recruitment efforts in predominately minority communities.

## Conclusion

The multi-dimensional, targeted promotion was critical for participation goals. Successful communication outreach is more effective through repeated exposure to messages in different ways.^28^ Multiple exposures to the message will have an impact on the belief about scientific discovery.^17^ The IMC approach relies on various modes of communications promoting a centralized message that is targeted, customized, and purposeful to recruit and maintain participant engagement. The study provided insight into how the IMC approach achieved the recommended recruitment numbers for the MTM-based PA intervention among African American women. Unforeseen factors may require modifications to the recruitment and retention plan, researchers utilizing the IMC approach can leverage the impact of diverse communication modes to ensure study information remains continual and consistent.^6^ IMC offers to researchers the benefit of synergizing individual communication elements in a coordinated manner used to address niche audiences and develop cost-effective health communications programs for improving recruitment and retention efforts among minority populations.

## Acknowledgements

The authors acknowledge the staff and management at the Jackson State University Walter Payton Recreation & Wellness Center, and the RCMI Center for Health Disparities Research (U54 MD015929) at Jackson State University.

## Funding

This research received no specific grants from any funding agency, in the public, commercial, or not-for-profit sectors.

Institutional Review Board (Protocol #0092-17).

## Competing interests

The authors declare that they have no conflict of interest.

## Ethical approval

This research was approved by the Jackson State University

## Authors’ contributions

Conceptualization (TH, MS); Methodology: TH; Formal analysis and investigation: TH; Writing: TH, MS; Reviewing and editing: MS.
